# FIBP interacts with transcription factor STAT3 to induce EME1 expression and drive radioresistance in lung adenocarcinoma

**DOI:** 10.7150/ijbs.83134

**Published:** 2023-07-24

**Authors:** Yunhong Xu, Jun Li, Kuikui Zhu, Yulan Zeng, Jing Chen, Xiaorong Dong, Sheng Zhang, Shuangbing Xu, Gang Wu

**Affiliations:** 1 Cancer Center, Union Hospital, Tongji Medical College, Huazhong University of Science and Technology, Wuhan 430022, China.; 2 Institute of Radiation Oncology, Union Hospital, Tongji Medical College, Huazhong University of Science and Technology, Wuhan 430022, China.

**Keywords:** FIBP, STAT3, EME1, radioresistance, lung adenocarcinoma

## Abstract

Cancer cells inevitably develop radioresistance during lung adenocarcinoma radiotherapy. However, the mechanisms are incompletely clarified. In this study, we show that FIBP protein expression in lung adenocarcinoma tissues is upregulated and associated with worse overall survival. Functionally, we find that depletion of FIBP inhibits lung adenocarcinoma progression and radioresistance *in vitro* and *in vivo*. Moreover, we uncover that FIBP interacts with STAT3 to enhance its transcriptional activity, thereby inducing the expression of the downstream target gene EME1. Importantly, we demonstrate that the biological effects of FIBP are partially dependent on EME1 in lung adenocarcinoma. Our work reveals that FIBP modulates the STAT3/EME1 axis to drive lung cancer progression and radioresistance, indicating that targeting FIBP may be a novel intervention strategy for lung adenocarcinoma radiotherapy.

## Introduction

Non-small cell lung cancer (NSCLC) accounts for approximately 85% of all lung cancer cases and lung adenocarcinoma is the major subtype of NSCLC 1, 2. Currently, the main treatments for lung cancer are surgery, chemoradiotherapy, targeted therapy and immunotherapy 3. Among these treatments, radiotherapy plays a pivotal role in lung cancer treatment at different stages. However, cancer cells inevitably develop radioresistance, leading to radiotherapy failure. Therefore, exploring novel targets and elucidating the mechanisms of radioresistance are important research priorities in lung cancer radiotherapy.

FGF1 intracellular binding protein (FIBP) is an acidic fibroblast growth factor (aFGF)-binding protein that mediates the action of aFGF 4, 5. FIBP gene mutation has been documented to be related to overgrowth syndrome and various congenital malformations 6, 7. In addition, knockout of FIBP can limit cholesterol metabolism and enhance the T cell antitumor efficacy 8. We previously reported that FIBP forms a complex with CDK5 and CDP138 that participates in the control of breast cancer cell growth and migration 9. However, the function and mechanism of FIBP in lung adenocarcinoma are totally unknown.

Signal transducer and activator of transcription 3 (STAT3) is involved in numerous biological processes, including cell apoptosis and proliferation, metastasis, angiogenesis, immunosuppression and drug resistance 10-13. Under normal conditions, STAT3 localizes to the cytoplasm and is then activated by phosphorylation at serine (727) and tyrosine (705) residues in response to different stimuli 14. Phosphorylation of STAT3 facilitates its nuclear translocation and binding to the promoters of target genes, thereby regulating their expression 14. In human cancers, STAT3 is generally hyperactivated and drives tumorigenesis, thus acting as an oncoprotein 15-17. In addition, accumulating evidence has revealed that the STAT3 signaling pathway modulates DNA damage repair 18, 19, supporting the roles of STAT3 in cancer radioresistance.

In this work, we report that FIBP is upregulated and contributes to lung adenocarcinoma progression and radioresistance *in vitro* and *in vivo*. We also find that FIBP binds to STAT3 to stimulate its transcriptional activity, inducing the expression of the target gene EME1. Moreover, the effects induced by FIBP silencing are partially dependent on EME1 in lung adenocarcinoma.

## Materials and Methods

### Cell culture

All cell lines were obtained from the American Type Culture Collection (ATCC) and cultured in RPMI 1640 medium or DMEM containing 10% fetal calf serum in 5% CO_2_ at 37°C. These cell lines were authenticated by short tandem repeat (STR) profiling and tested for mycoplasma contamination. Plasmid transfection was conducted using polyethyleneimine or Lipofectamine 200 (Invitrogen, Camarillo, CA, USA).

### Antibodies

The anti-FIBP (ab236647), anti-Rad51 (ab133534) and anti-γH2AX (phospho S139) (ab26350) antibodies were purchased from Abcam (Cambridge, MA, UK). The anti-GST (#2624), anti-phospho-STAT3 (Ser727, #9134), anti-phospho-STAT3 (Tyr705, #9131) and anti-STAT3 (#9139) antibodies were obtained from Cell Signaling Technology (Danvers, MA, USA). The anti-EME1 (sc-393363) antibody was obtained from Santa Cruz Biotechnology (Santa Cruz, CA, USA). The anti-Flag (AE063), anti-Myc (AE07), anti-HA (AE008), and anti-GAPDH (A19056) antibodies were purchased from ABclonal (Wuhan, China).

### siRNA interference (RNAi)

Lipofectamine RNAiMAX (100 nM; Invitrogen) was used for siRNA transfection into cancer cells. Cells were harvested after 48 h for analysis. The sequences of the FIBP siRNAs were as follows:

Si-FIBP#1, 5′-GGUGGACAAUAUUCAGCAA-3′; Si-FIBP#2, 5′-CCAUCGUCUUCUUUGCUAA-3′.

### Lentivirus infection

Lentiviral particles were generated by cotransfection of FIBP shRNAs with pMD2.G and psPAX2 into HEK293T cells. The supernatants were collected after 48 h and filtered to infect A549 cells in the presence of 10 µg/mL polybrene. Puromycin (2 μg/mL) was used to screen the transduced cells for seven days, and knockdown of FIBP was confirmed by western blot analysis. The following FIBP shRNAs were used: Sh-FIBP#1, 5′-TGCTGAATATTGTCCACCA-3′; Sh-FIBP#2, 5′-TTGGTGCTGATGTCATCCA-3′ 9.

### Western blot analysis and immunoprecipitation

Cells were lysed in NETN lysis buffer (20 mM Tris-HCl [pH 8.0], 100 mM NaCl, 1 mM EDTA and 0.5% Nonidet P-40), and the supernatant was boiled at 100°C for 10 min. Proteins in the samples were separated by SDS-PAGE, and immunoblotting was conducted using the indicated antibodies. For immunoprecipitation, cell lysates were incubated with S-protein agarose (Novagen, Madison, WI, USA) or primary antibodies plus protein A/G agarose (Santa Cruz Biotechnology) and were then analyzed by western blotting.

### GST pull-down assay

Purified GST or GS​​T-STAT3 proteins were immobilized on Glutathione-Sepharose 4B beads (GE Healthcare) and incubated with lysates of cells transfected with SFB-FIBP overnight at 4°C. The immunoprecipitates were washed and analyzed by immunoblotting.

### RNA sequencing (RNA-seq)

H1299 cells were transfected with scrambled or FIBP-targeting siRNAs for 48 h, and total RNA was isolated. Genes that met the threshold criteria of fold change > 2.0 and false discovery rate (FDR) < 5% were considered significantly differentially expressed. The RNA-seq data have been deposited in the Gene Expression Omnibus (GEO) database under accession number GSE196635.

### Real-time quantitative PCR

TRIzol reagent was used to isolate total RNA from cancer cells, and RT SuperMix was used for reverse transcription. Real-time quantitative PCR was conducted using a SYBR Green qPCR Master Mix. The primer sequences are listed in Supplemental Table S1.

### Luciferase reporter assay

Cells were collected, seeded in a 12-well plate, and cotransfected with the pSTAT3-TA-Luc and Renilla plasmids. After 24 h, a Dual-Lumi™ II Luciferase Reporter Gene Assay Kit (Promega) was used to measure the intensity of luciferin.

### ChIP Assay

ChIP assay was performed following the protocol of the EZ-ChIP TM Chromatin immune-precipitation kit (Millipore). Briefly, 5 × 10^6^ cells were incubated in 1% formaldehyde for 10 min at 37 °C, glycine was added and incubated for 5 min at room temperature to terminate the crosslink reaction. Cell were lysed and sheared by sonication to get 200-400 bp chromatin fragment. After precleared, chromatin fragment was incubated with anti-STAT3 antibody and immunoprecipitated by protein A /G plus agarose. DNA from eluted chromatin was amplified by PCR using gene promoter-specific primers. Data were normalized to input control. The ChIP primer sequences are listed in Supplemental Table S2.

### 5-Ethynyl-2'-deoxyuridine (EdU) incorporation assay

An EdU Kit with Alexa Fluor 594 was obtained from Beyotime, and the EdU incorporation assay was performed according to the manufacturer's protocol. After cell fixation and membrane permeabilization, EdU was labeled using Alexa Fluor 594. Images were acquired by fluorescence microscopy and were then analyzed.

### Cell cycle analysis

Cells were treated and irradiated (6 Gy). After 24 h, the cells were harvested and fixed with 70% ethanol overnight. After three washes with cold PBS, the cells were resuspended in DNase-free RNase A buffer (50 µg/ml) for 30 min, stained with propidium iodide (50 µg/ml), and subjected to FACS analysis.

### Carboxy fluorescein succinimidyl ester (CFSE) assay

Cells were stained with CFSE for 15 min and the CFSE-stained cells were then seeded into six-well plates. At different times, the cells were tyrosinated, harvested and analyzed by FACS.

### Clonogenic survival assay

After digestion and centrifugation, cells were resuspended as single cells in culture medium, seeded into a 6-well plate and irradiated. After two weeks, colonies were fixed and stained, and colonies containing more than 50 cells were counted. The survival rates of the groups subjected to different radiation treatments were calculated.

### Neutral comet assay

The neutral comet assay was conducted according to the manufacturer's protocol (Trevigen). In brief, after 4 h of exposure to 6 Gy irradiation, cells were trypsinized, washed, and resuspended. The cells were then mixed with 37°C low-melting point agarose and layered onto pretreated grass slides. The slides were immersed in lysis buffer for 1 h and incubated in precooled running buffer for electrophoresis at 21 V for 60 min. The slides were soaked and stained with SYBR Gold for 10 min, and images were then acquired and analyzed.

### Immunofluorescence staining

Cells were seeded on glass slides. After 4 h of radiation exposure (6 Gy), the cells were fixed, permeabilized and blocked with 5% goat serum albumin. After incubation with primary antibodies overnight, the cells were washed and incubated with Dylight549- or 488-conjugated secondary antibodies for 1 h. The cells were stained with 4',6-diamidino-2-phenylindole (DAPI) and observed using a confocal microscope.

### Tissue microarray and immunohistochemical (IHC) staining

A human adenocarcinoma tissue microarray containing 82 paired lung carcinoma tissues was provided by Shanghai Outdo Biotech. IHC analysis was conducted as previously described 20, 21. The IHC score (based on the cellular staining intensity and the number of positive cells) was used to evaluate the expression of FIBP in each stained tissue.

### Mouse xenograft model

All animal experiments were approved by the Medical Ethics Committee of Tongji Medical College, Huazhong University of Science and Technology. Five-week-old BALB/c nude mice were randomly grouped, and the experiment was carried out following the animal centre's operating procedure. A xenograft model was established by subcutaneous injection of 4 ×10^6^ control or FIBP knockdown A549 cells. For the irradiation experiment, mice were irradiated with 10 Gy when the tumor volume had increased to about 130 mm^3^. The tumor size was evaluated every three days, and the tumor volume was calculated according to the formula (L × W^2^)/2.

### Statistical analysis

All experiments were repeated independently at least three times, and all data are presented as the mean ± SD values unless otherwise indicated. The significance of differences between the two independent groups was analyzed by two-tailed Student's t test. P < 0.05 was considered significant.

## Results

### The FIBP protein is overexpressed and correlated with poor outcomes in lung adenocarcinoma

**Fig 1:**
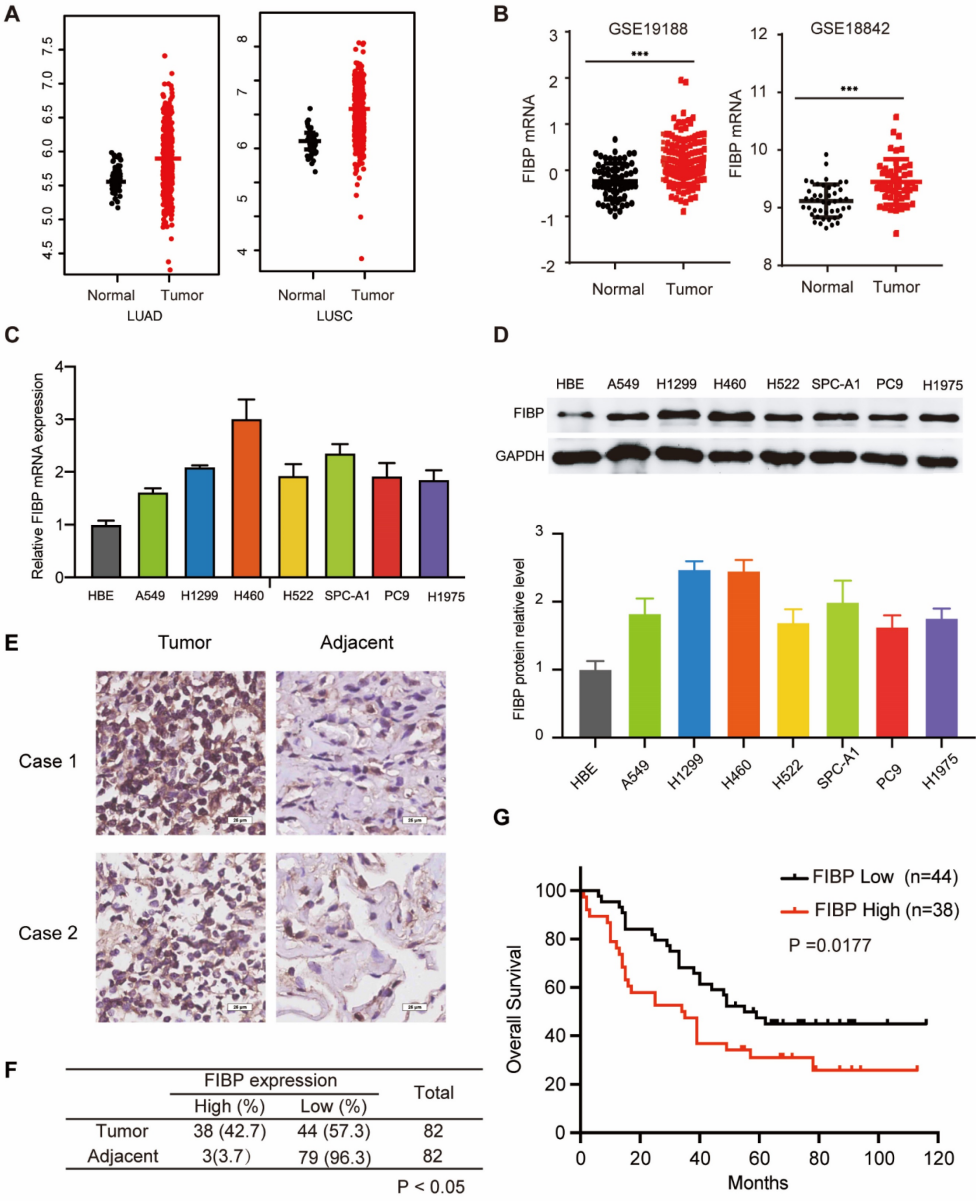
** FIBP is upregulated in lung adenocarcinoma and associated with poor outcomes. (A-B)** Analysis of FIBP mRNA expression in normal and tumor tissues from the TCGA and GEO lung adenocarcinoma datasets. **(C-D)** PCR and Western blot analysis of FIBP in human bronchial epithelial (HBE) cells and different lung adenocarcinoma cell lines.** (E)** Images of FIBP IHC staining in the lung adenocarcinoma tissue microarray. Scale bar, 25 μm. **(F)** Statistical analysis of FIBP IHC results. **(G)** High FIBP expression was associated with worse outcomes in lung adenocarcinoma patients.

To explore the mRNA level of FIBP in lung adenocarcinoma, we first analyzed matched NSCLC samples in The Cancer Genome Atlas (TCGA) and GEO and found that the mRNA level of FIBP was increased in lung cancer tissues compared with normal tissues (Fig. 1A-B and Fig. S1A). Furthermore, we showed that the FIBP mRNA and protein levels were higher in lung adenocarcinoma cell lines than in the normal cell line HBE (Fig. 1C-D). We then quantified the protein level of FIBP in a human lung adenocarcinoma tissue microarray. As shown in Fig. 1E-F and Fig. S1B, the FIBP protein levels were increased in the lung cancer tissues compared with the paired adjacent lung tissues. In addition, patients with high FIBP expression had shorter overall survival (Fig. 1G). Thus, our results indicate that FIBP may function as an oncoprotein in lung adenocarcinoma.

### FIBP depletion impairs lung adenocarcinoma progression *in vitro* and *in vivo*

**Fig 2:**
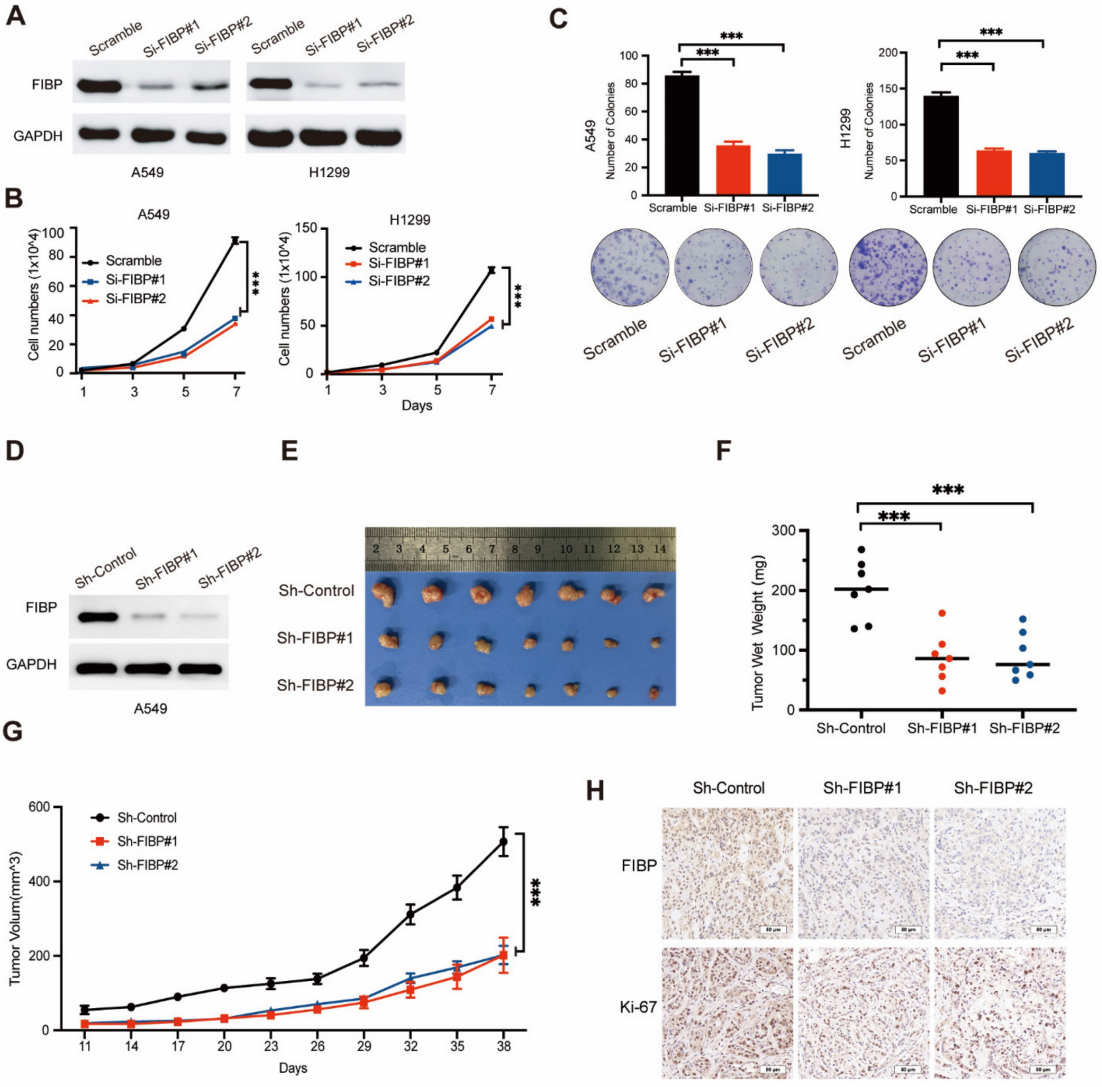
** FIBP knockdown suppresses lung adenocarcinoma cell growth and proliferation *in vitro* and* in vivo*. (A)** Immunoblot analysis of FIBP protein expression in A549 and H1299 cells.** (B-C)** FIBP depletion inhibited the growth and proliferation of A549 and H1299 cells. ***P < 0.001. **(D)** FIBP was stably depleted by two different shRNAs (Sh-FIBP) in A549 cells. **(E)** Pictures of xenograft tumors (n = 7 mice/group). **(F)** FIBP silencing suppressed tumor growth* in vivo*. The data are presented as the mean ± SEM values. ***P < 0.001. **(G)** Tumor weights in the three groups. ***P < 0.001. **(H)** Pictures of FIBP and Ki-67 staining of xenograft tumors.

Given that FIBP is upregulated in lung adenocarcinoma tissue, we hypothesized that FIBP may contribute to tumorigenesis. To test this hypothesis, two different siRNAs were used to knock down FIBP expression in H1299 and A549 cells (Fig. 2A). Downregulation of FIBP significantly reduced the growth and proliferation ability of lung cancer cells (Fig. 2B-C). In addition, the CFSE proliferation assay showed that FIBP depletion inhibited cell proliferation (Fig. S2A).Consistent with this finding, the proportions of EdU-positive cells and S phase cells were significantly reduced in the FIBP knockdown group (Fig. S2B-C). Collectively, these data support the hypothesis that FIBP drives lung adenocarcinoma cell proliferation *in vitro*.

To further confirm this phenomenon *in vivo*, we generated FIBP knockdown cancer cell lines stably expressing FIBP-targeting shRNAs (Fig. 2D). Consistent with the *in vitro* results, mice injected with FIBP knockdown cells had reduced tumor sizes and weights compared with those of mice injected with control cells (Fig. 2E-H). These results strongly demonstrate that FIBP silencing impairs lung adenocarcinoma progression *in vitro* and *in vivo*.

### Suppression of FIBP expression enhances lung adenocarcinoma radiosensitivity *in vitro* and* in vivo*

**Fig 3:**
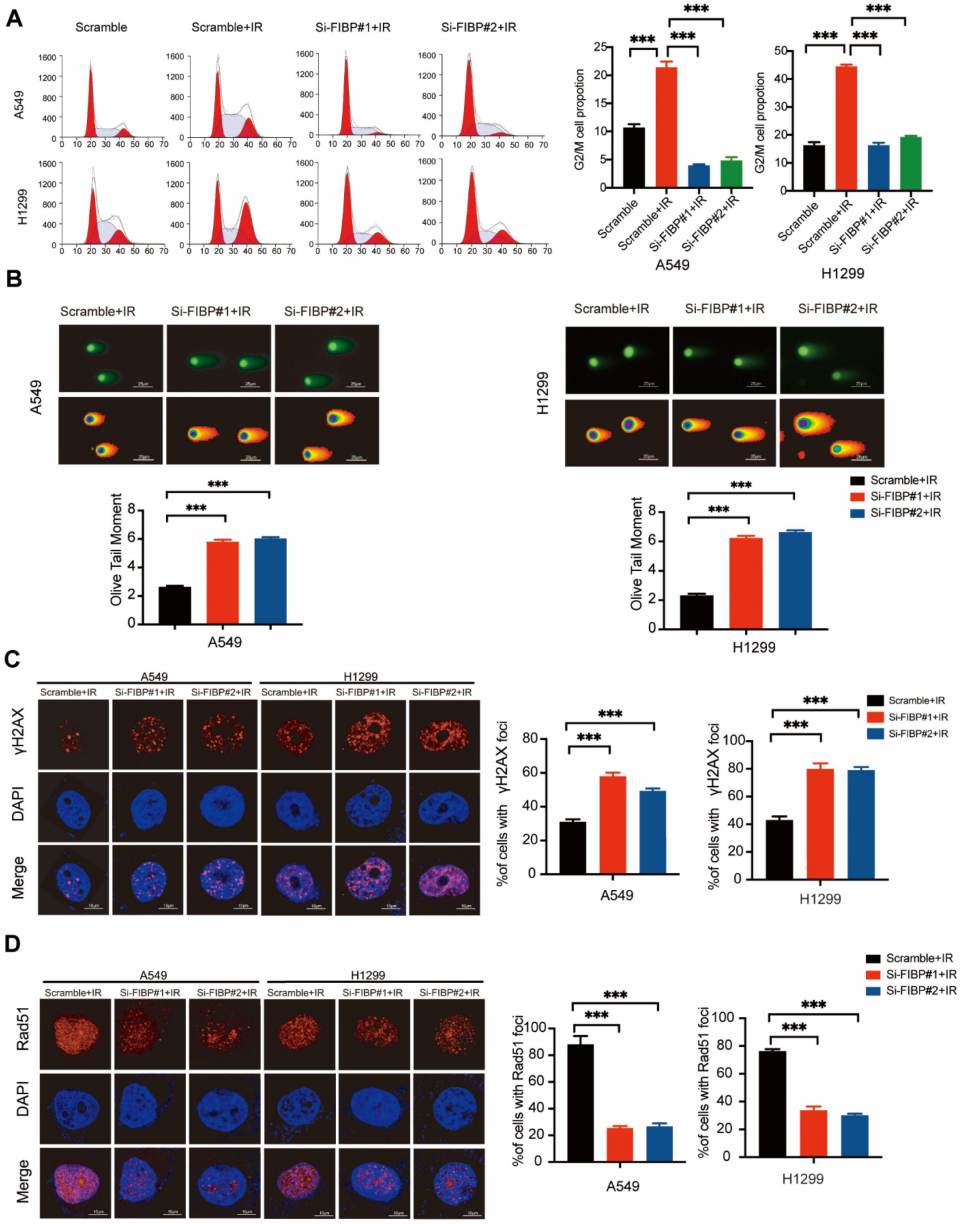
** FIBP depletion enhances DNA damage response and inhibits DNA repair in lung adenocarcinoma. (A)** Cells were transfected with indicated FIBP siRNAs for 48 h and exposed to IR. After 24 h, cells were collected and analyzed by flow cytometry. ***P < 0.001. **(B)** Representative images and quantification of the Olive tail moment in control cells and FIBP-depleted lung adenocarcinoma cells. ***P < 0.001. **(C)** Left panel: Control cells and FIBP-depleted lung adenocarcinoma cells were irradiated and were then harvested after 4 h. Representative images of γH2AX foci. Right panel: quantification of γH2AX foci. ***P < 0.001. Scale bar, 10 μm. **(D)** FIBP depletion impaired Rad51 foci formation. ***P < 0.001. Scale bar, 10 μm.

After exploring the function of FIBP under normal conditions, we also investigated the phenotypes caused by FIBP knockdown in response to irradiation. As shown in Fig. 3A, irradiation-induced G2/M cell cycle arrest was significantly abolished in FIBP-depleted cells. Furthermore, cells transfected with FIBP siRNA exhibited a longer Olive tail moment, indicating an increase in DNA double-strand breaks (Fig. 3B). Consistent with this finding, the proportion of γ-H2AX foci-positive cells was increased significantly when FIBP was depleted (Fig. 3C). Next, we performed a Rad51 foci formation assay and showed that Rad51 foci formation was impaired in FIBP-deficient cells (Fig. 3D), suggesting that FIBP knockdown inhibits DNA repair.

**Fig 4:**
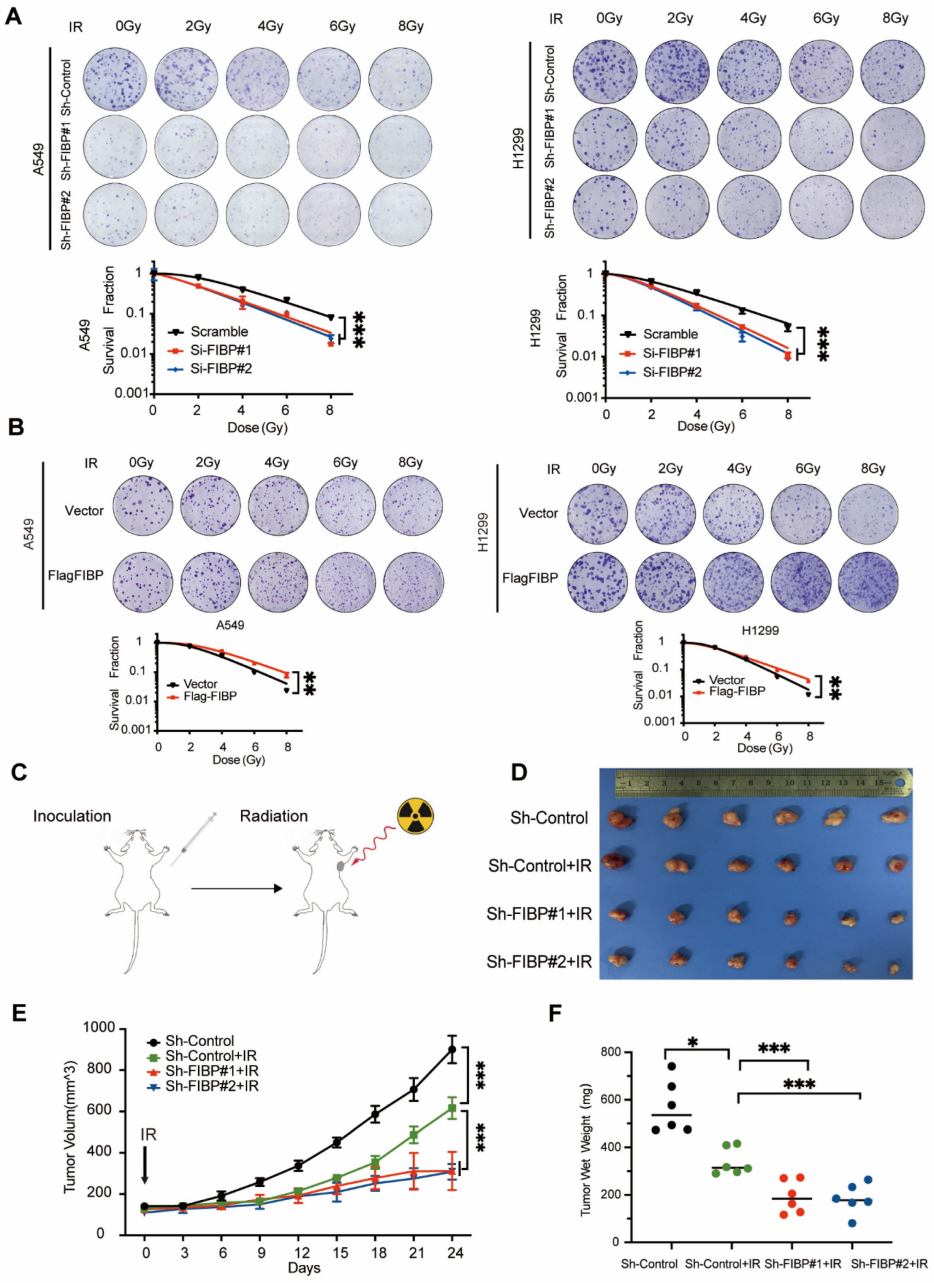
** FIBP depletion contributes to enhanced radiosensitivity in lung adenocarcinoma *in vitro* and* in vivo*. (A)** The cell survival rate showed that FIBP depletion sensitized lung cancer cells to radiation. ***P < 0.001. **(B)** FIBP overexpression led to radioresistance in lung adenocarcinoma cells. **P < 0.01. **(C)** Mice were irradiated with 10 Gy when the tumor volume had increased to about 130 mm^3^. **(D-F)** Images of xenograft tumors (D), tumor growth curves (E) and tumor weight curves (F) in different groups (n = 6 mice/group). *P < 0.05, ***P < 0.001.

Moreover, loss of FIBP led to enhanced radiosensitivity in lung adenocarcinoma cells, whereas overexpression of FIBP exhibits reverse effect (Fig. 4A-B). These results indicate that FIBP indeed promotes lung adenocarcinoma radioresistance *in vitro*. Next, we established a xenograft mouse model *in vivo*. As shown in Fig. 4C-E, tumor growth was slowed in nude mice that received radiation treatment, indicating that radiation alone was effective. More importantly, the tumor sizes and weights were significantly reduced in the group treated with Sh-FIBP combined with irradiation (Fig. 4E-F). Collectively, our data show that loss of FIBP enhances lung adenocarcinoma radiosensitivity *in vitro* and *in vivo*.

### FIBP silencing decreases the mRNA and protein levels of EME1 in lung adenocarcinoma cells

**Fig 5:**
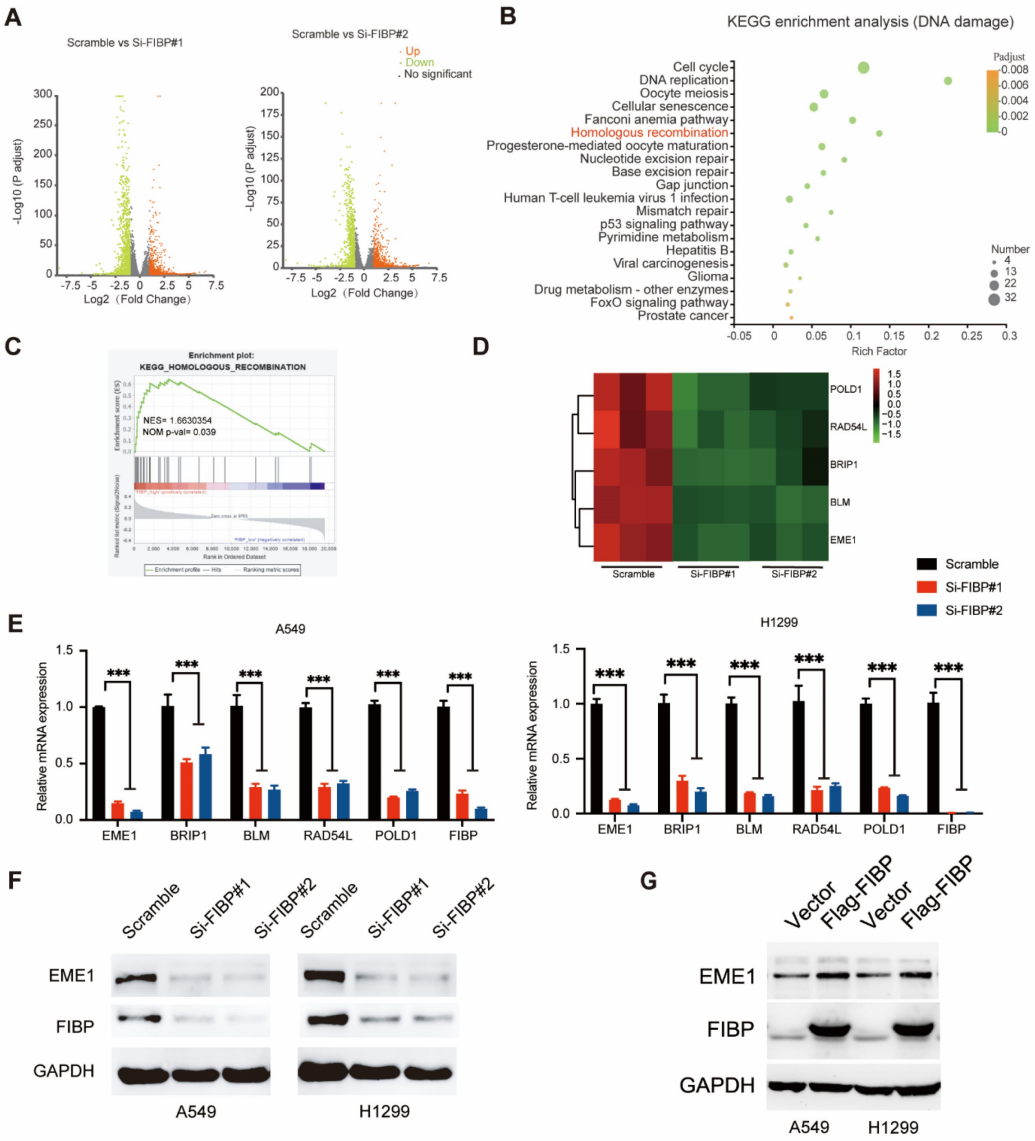
** Suppression of FIBP downregulates EME1 expression in lung adenocarcinoma cells. (A)** Volcano plot showing up- and downregulated genes in FIBP-depleted H1299 cells. **(B)** Kyoto Encyclopedia of Genes and Genomes (KEGG) pathway enrichment analysis of transcriptionally altered genes, as identified by RNA-seq, between control cells and FIBP-depleted lung adenocarcinoma cells. **(C)** The pathway enrichment analysis shows that FIBP is closely correlated with homologous recombination. **(D)** Heatmap of the transcriptional changes. **(E)** Real-time PCR analysis showed the changes in mRNA expression in H1299 cells. ***P < 0.001.** (F-G)** FIBP positively regulates EME1 protein expression in lung adenocarcinoma cells.

To identify the downstream effector of FIBP in lung adenocarcinoma, we used RNA-seq to explore the transcription profiles of lung adenocarcinoma cells transfected with FIBP siRNAs. As shown in Fig. 5A, many genes were differentially expressed in FIBP-depleted cells. Further pathway enrichment analysis revealed that FIBP was closely related to homologous recombination (Fig. 5B-C). We then validated five genes involved in homologous recombination using real-time PCR (Fig. 5D-E). Among these genes, EME1 exhibited the greatest downregulation in FIBP-depleted lung cancer cells (Fig. 5E). EME1 is a catalytic subunit that interacts with Mus81 to form a structure-specific endonuclease to maintain genomic stability in mammalian cells 22-24. Notably, loss of FIBP also led to decreased protein levels of EME1 in two different lung adenocarcinoma cell lines, whereas overpression of FIBP increased the expression of EME1 (Fig. 5F-G). In addition, this correlation between FIBP and EME can also be observed in the animal model (Fig. S3). Taken together, our results indicate that FIBP regulates both the transcription and translation of EME1.

### FIBP interacts with STAT3 to induce the expression of EME1 in lung adenocarcinoma cells

**Fig 6:**
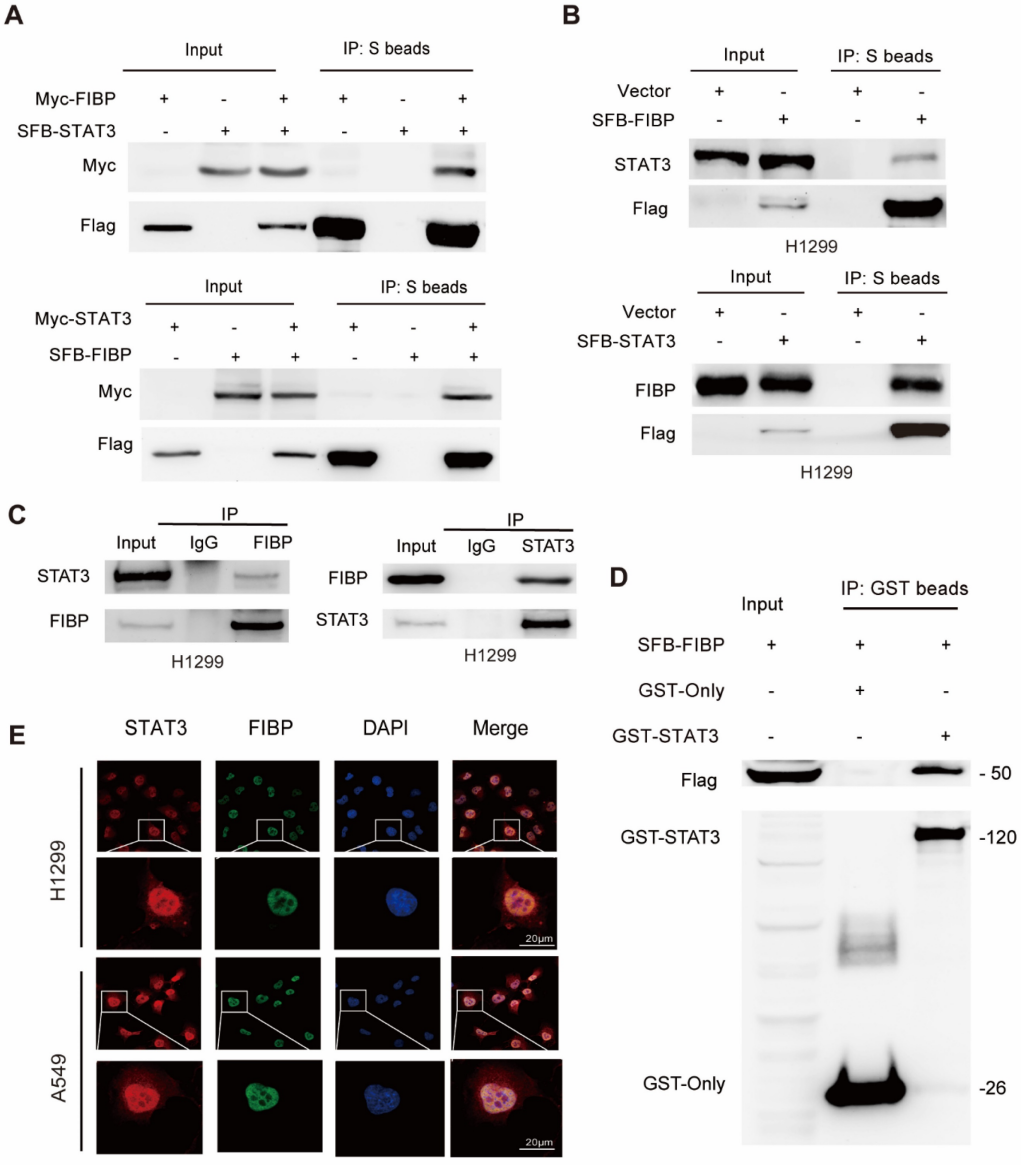
** FIBP interacts with STAT3 in lung adenocarcinoma cells. (A)** Exogenously expressed STAT3 bound to Exogenously expressed FIBP and vice versa in H1299 cells. **(B)** Exogenously expressed STAT3 formed a complex with endogenous FIBP and vice versa in H1299 cells.** (C)** Endogenous binding between FIBP and STAT3 in H1299 cells. **(D)** The GST or recombinant GST-STAT3 protein was coimmunoprecipitated with SFB-FIBP overnight. The immunoprecipitates were analyzed by western blotting. **(E)** Colocalization of endogenous FIBP and STAT3 in H1299 cells.

STAT3 is well documented to be able to modulate the transcription of EME1 25, 26. We therefore surmised that FIBP may control the expression of EME1 via STAT3. To this end, we first detected the interaction between FIBP and STAT3. As expected, exogenously expressed STAT3 formed a complex with exogenous or endogenous FIBP and vice versa (Fig. 6A-B). A clear endogenous interaction between FIBP and STAT3 was also observed in lung adenocarcinoma cells (Fig. 6C). Furthermore, we demonstrated that recombinant GST-tagged STAT3 interacted with FIBP (Fig. 6D). Immunofluorescence staining showed that endogenous FIBP colocalized with STAT3 in the nucleus (Fig. 6E). These results support that FIBP associates with STAT3 in lung adenocarcinoma cells.

**Fig 7:**
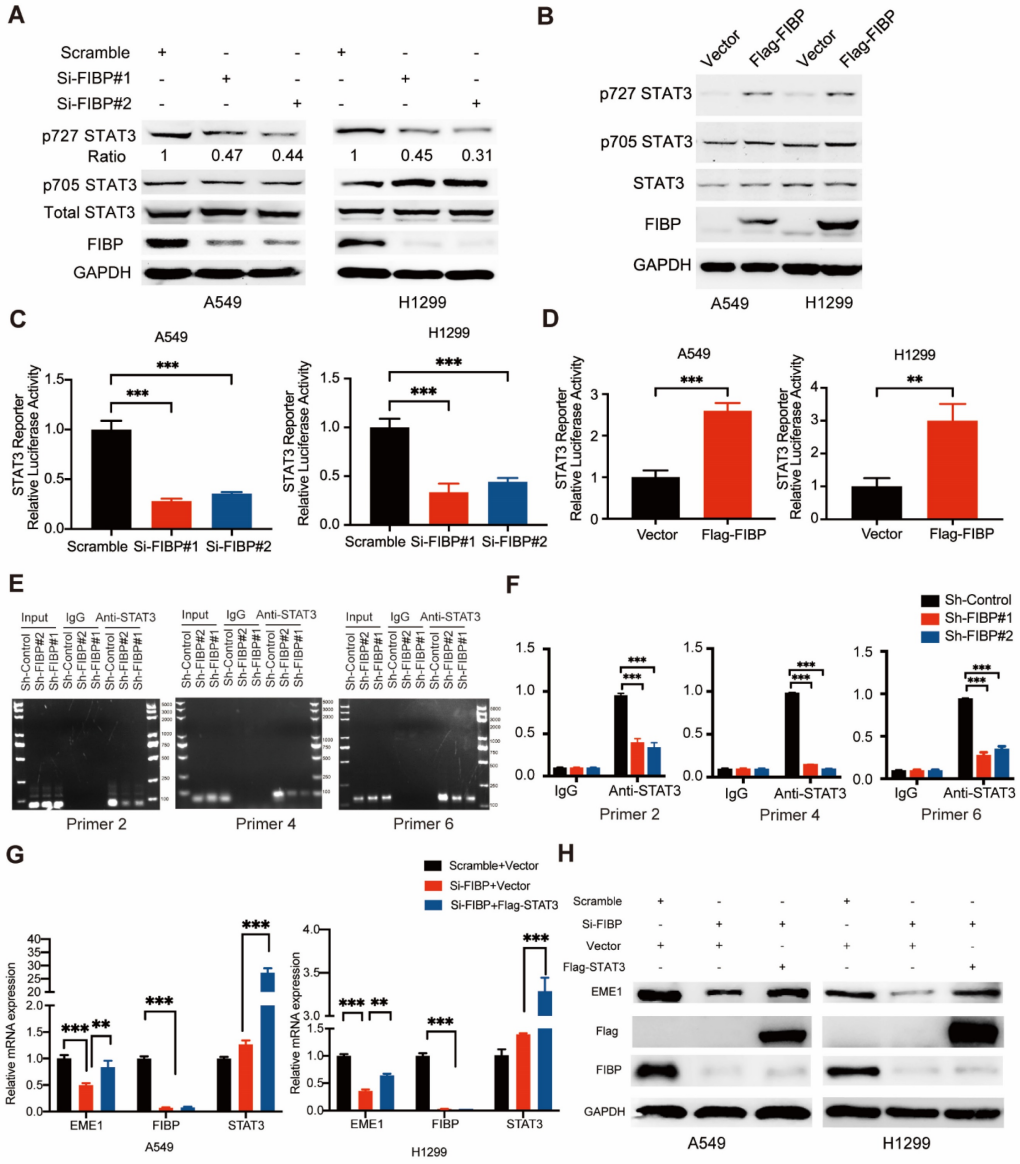
** FIBP enhances STAT3 transcriptional activity to induce EME1 expression in lung adenocarcinoma cells. (A-B)** FIBP positively regulated the phosphorylation of STAT3 at Ser727 in lung adenocarcinoma cells. **(C-D)** The luciferase reporter assay showed that FIBP positively regulates the transcriptional activity of STAT3 in A549 and H1299 cells. **P < 0.01, ***P < 0.001. **(E-F)** FIBP depletion decreased the binding affinity of STAT3 to EME1 gene promoter. ***P < 0.001.** (G-H)** PCR and Western blot analysis of the expression of EME1 in lung adenocarcinoma cells after transfection with the indicated plasmids or siRNAs.

Given that FIBP binds to STAT3, a well-known transcription factor, we speculated that FIBP might control STAT3 transcriptional activity. As shown in Fig. 7A-B, FIBP silencing downregulated the phosphorylation of STAT3 at Ser727 and FIBP overexpression increased the phosphorylation of STAT3 at Ser727, while the levels of STAT3 phosphorylated at Tyr705 and total STAT3 were not altered. Consistent with this result, luciferase reporter assays showed that knockdown of FIBP inhibited but overexpression of FIBP enhanced STAT3 transcriptional activity (Fig. 7C-D). To elucidate whether FIBP can control the affinity of STAT3 in the promoter region of EME1, we performed the ChIP PCR and showed that FIBP silencing decreased the binding affinity of STAT3 to EME1 gene promoter (Fig. 7E-F).

Since EME1 is a target gene of STAT3, we next asked whether FIBP controls the expression of EME1 via STAT3. As shown in Fig. 7G-H, overexpression of STAT3 partially reversed the decrease in the mRNA and protein levels of EME1 in FIBP-deficient lung adenocarcinoma cells, indicating that FIBP controls the expression of EME1 in a STAT3-dependent manner. Accordingly, these results suggest that FIBP binds to STAT3 to enhance its transcriptional activity, thereby inducing EME1 expression in lung adenocarcinoma cells.

### The biological effects of FIBP inhibition are mediated partially via modulation of EME1 in lung adenocarcinoma cells

**Fig 8:**
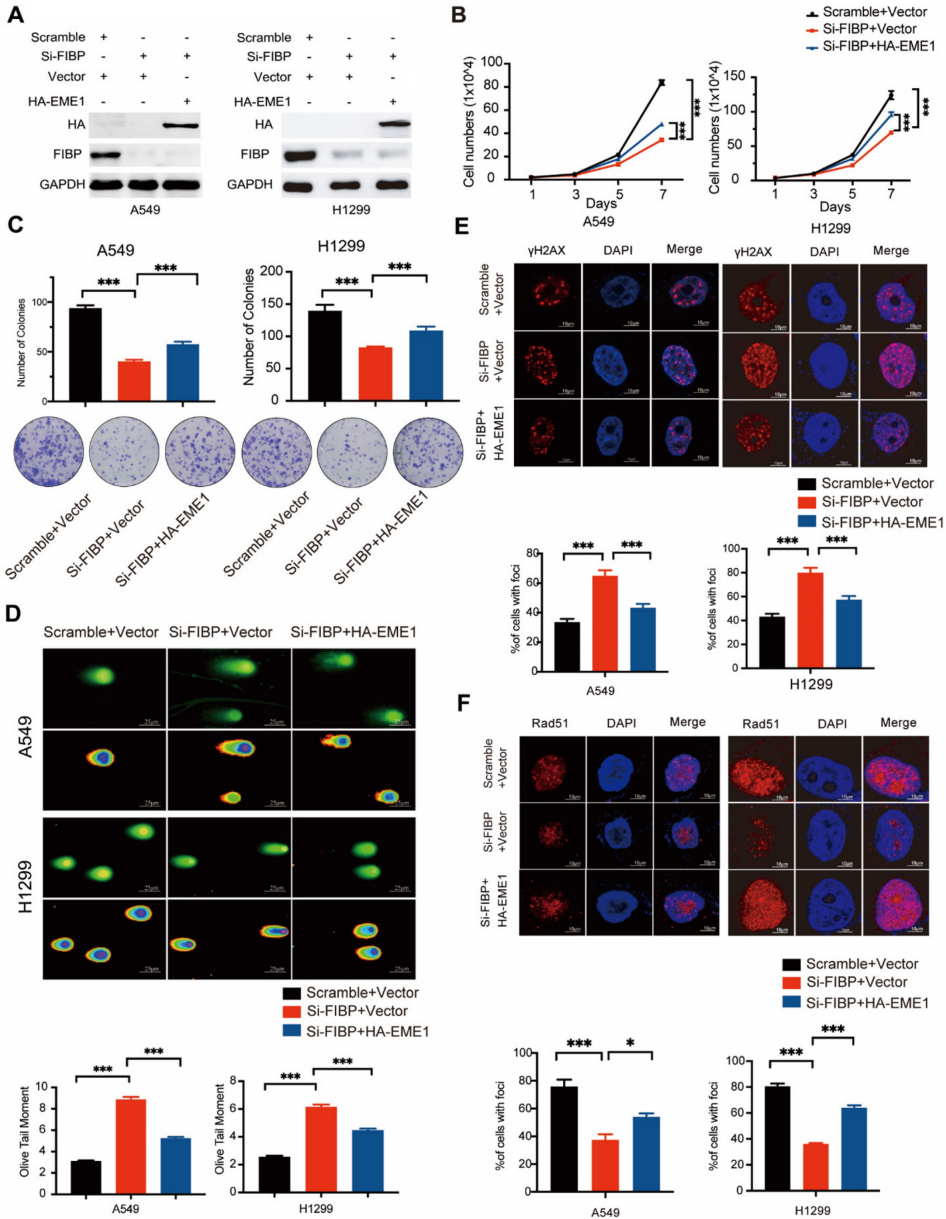
** The biological effects of FIBP inhibition are mediated partially by modulating EME1 in lung adenocarcinoma cells. (A)** Immunoblot analysis of the indicated proteins in H1299 cells. **(B-C)** Cell growth (B) and colony formation (C) assays showed that the ability of FIBP to inhibit cell growth and proliferation was abrogated by overexpression of EME1 in H1299 cells. ***P < 0.001. **(D)** Representative images and quantification of the Olive tail moment indicating DNA damage induced by irradiation. ***P < 0.001. **(E-F)** Representative immunofluorescence images and quantification of γH2AX foci (E) and Rad51 foci (F) in H1299 cells. *P < 0.05, ***P < 0.001.

To determine whether EME1 is an effector of FIBP in lung adenocarcinoma, we transfected the EME1 plasmid into FIBP-deficient H1299 cells (Fig. 8A). As illustrated in Fig. 8B-C, reconstitution of EME1 partially reversed the suppressive effects of FIBP knockdown on lung cancer cell growth and proliferation. Moreover, FIBP-deficient lung cancer cells showed increases in γ-H2AX foci formation and the Olive tail moment, and these increases were partially reversed by overexpression of EME1 (Fig. 8D-E). Consistent with the above results, overexpression of EME1 partially reversed the decrease in Rad51 foci formation in FIBP-deficient lung cancer cells (Fig. 8F). These data support that FIBP participates in lung adenocarcinoma cell proliferation and radioresistance by upregulating EME1.

## Discussion

Radiotherapy is usually accompanied by the development of radioresistance in lung adenocarcinoma cells, which can lead to local recurrence and poor prognosis. Here, we revealed that FIBP associates with STAT3 to stimulate its transcriptional activity, which consequently induces the expression of EME1, thereby driving lung adenocarcinoma radioresistance. In addition, we showed that FIBP is overexpressed in lung cancer and that high FIBP expression is correlated with worse survival, implying that FIBP may be a promising prognostic marker in lung adenocarcinoma.

As STAT3 is a key transcription factor, its activity is finely modulated by its interaction with various proteins 27-30. For instance, ApoC1 has been reported to interact with STAT3 and enhance its phosphorylation level, whereas FBP1 binds to STAT3 and represses PD-L1 expression 29, 30. In our work, we identified FIBP as a novel STAT3-interacting protein in non-small cell lung cancer. This interaction led to increased phosphorylation of STAT3 and stimulated its transcriptional activity. In contrast, depletion of FIBP markedly decreased STAT3 phosphorylation and activation, thereby inhibiting the transcription of EME1. We further showed that overexpression of exogenous STAT3 reversed the decrease in EME1 expression in FIBP-deficient lung cancer cells, indicating that FIBP upregulates EME1 in a STAT3-dependent manner. Taken together, these findings demonstrate that FIBP is a novel positive regulator of STAT3 and controls STAT3-dependent EME1 expression in lung adenocarcinoma.

STAT3 has been implicated in inflammation, cell growth, antitumor immunity and radioresistance in a variety of human malignancies 31-33. For example, depletion of STAT3 stimulates T-cell activity and enhances radiosensitivity in head and neck cancer 34. In addition, BMX inhibition inactivates STAT3 to reduce radioresistance in glioma 35. In our study, we found that FIBP bound to STAT3 and enhanced its transcriptional activity, indicating that FIBP may function in tumorigenesis and radioresistance. Indeed, we discovered that FIBP was upregulated and predicted worse outcomes in lung adenocarcinoma. Further functional studies clearly showed that knockdown of FIBP significantly suppressed tumorigenesis, supporting the oncogenic role of FIBP in lung cancer. Furthermore, we unveiled a new function of FIBP in driving radioresistance in lung adenocarcinoma. Suppression of FIBP inhibited DNA damage repair and contributed to enhanced radiosensitivity in lung cancer cells. RNA-seq uncovered that EME1 was a downstream target gene of FIBP and that its mRNA and protein levels were controlled by FIBP. It has been documented that EME1 binds to Mus81 to recognize several DNA structures and plays essential roles in homologous recombination repair 22-24. Our data demonstrated that the observed effects induced by FIBP silencing were reversed at least partially by ectopic expression of EME1, implying that EME1 also participates in the biological function of lung adenocarcinoma. Therefore, these results support the notion that EME1 is a critical effector of FIBP, which plays important roles in lung adenocarcinoma radioresistance.

In summary, our research illustrates that FIBP interacts with STAT3 to increase its phosphorylation, thereby enhancing its transcriptional activity and inducing the expression of its target gene EME1, which ultimately contributes to lung adenocarcinoma progression and radioresistance. Our results not only elucidate the mechanism by which FIBP functions but also provide a novel promising target for lung adenocarcinoma radiotherapy.

## Supplementary Material

Supplementary figures and tables.
