# Supplementary material for: FIBP interacts with transcription factor STAT3 to induce EME1 expression and drive radioresistance in lung adenocarcinoma
Source: Int J Biol Sci. 2023 Jul 24;19(12):3816–29. doi: 10.7150/ijbs.83134 (PMC10411469; doi:10.7150/ijbs.83134)
Supplement: Supplementary file 1 — Supplementary figures and tables. [file ijbsv19p3816s1.pdf]

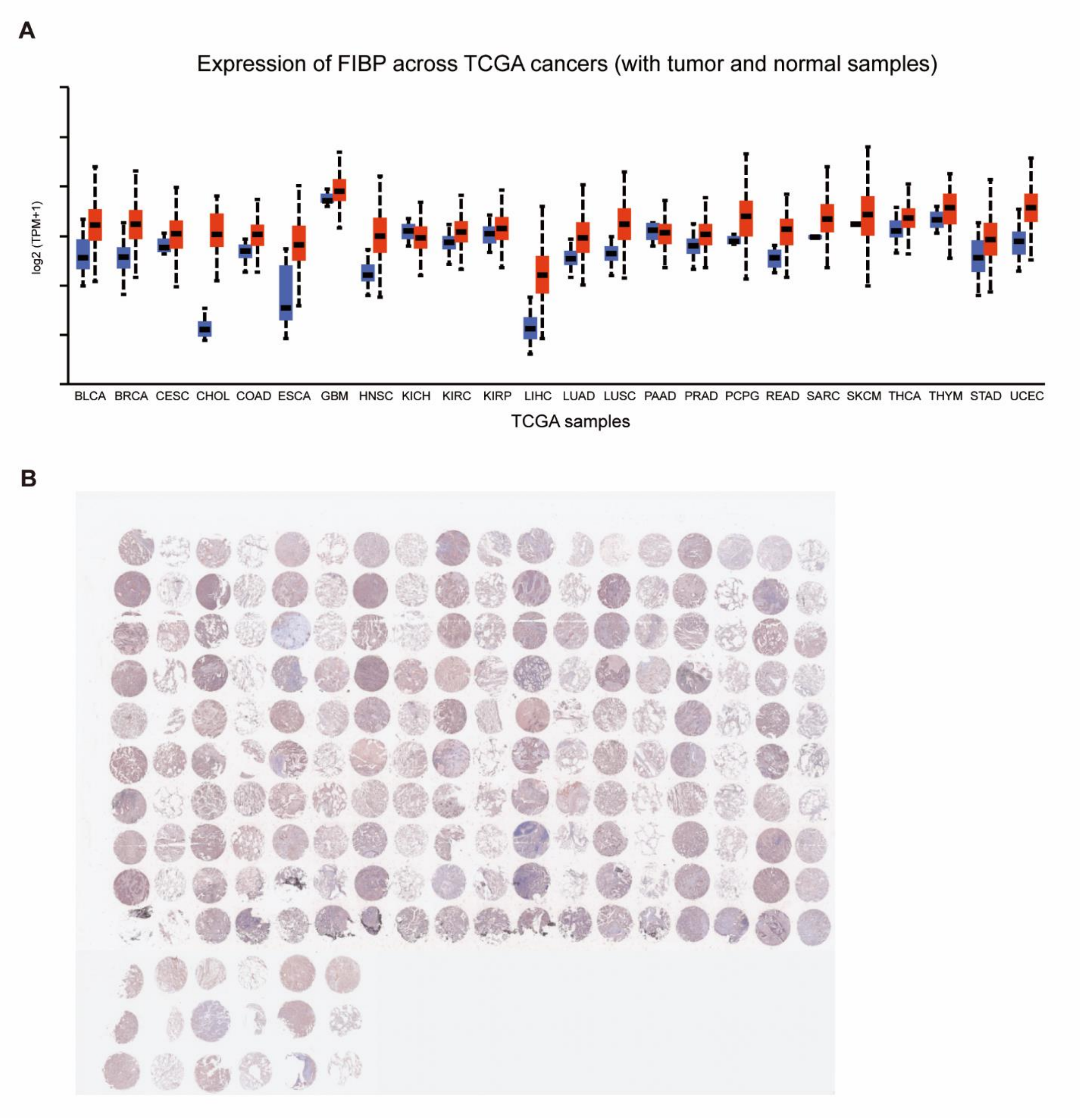


**Fig. S1. (A)** FIBP expression profile across multiple TCGA tumor and paired normal tissues. **(B)** FIBP protein levels in a human lung adenocarcinoma tissue microarray containing 82 paired lung adenocarcinoma and adjacent lung tissues.

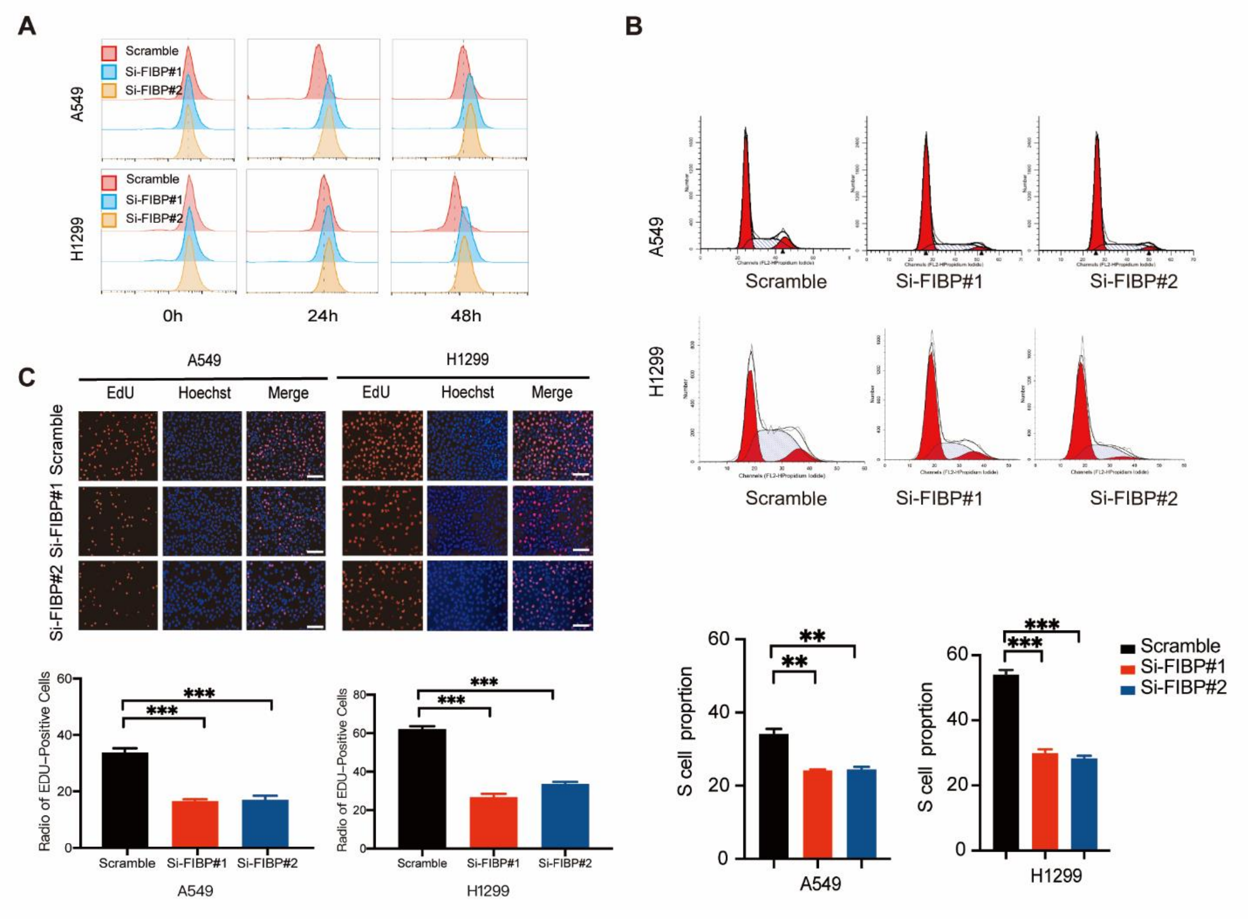


**Fig. S2. FIBP silencing inhibits non-small cell lung cancer cell proliferation. (A)** CFSE was used to evaluate the proliferation ability of lung cancer cells. **(B)** FIBP downregulation reduced the population of S phase cells. **P < 0.01, ***P < 0.001. **(C)** The percentage of EdU-positive cells in FIBP-depleted lung cancer cells was decreased. ***P < 0.001.

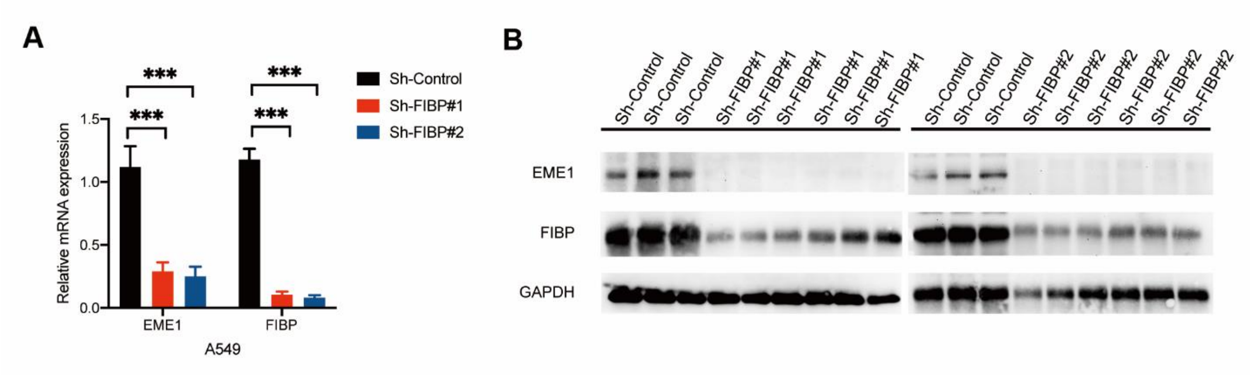


**Fig. S3. The mRNA and protein expression of FIBP and EME1 in the animal model. (A)** Real-time PCR analysis showed the mRNA expression of FIBP and EME in xenograft tumors (n = 6). ***P < 0.001. **(B)** Immunoblot analysis of FIBP and EME1 protein levels in xenograft tumors (n = 6).

**Supplemental Table S1.** Primers Sequences used for Real-time PCR

| Genes | Sequences (5'-3') |
|---|---|
| BLM | F: CAGACTCCGAAGGAAGTTGTATG |
| | R: TTTGGGGTGGTGTAACAAATGAT |
| BRIP1 | F: CTTACCCGTCACAGCTTGCTA |
| | R: CACTAAGAGATTGTTGCCATGCT |
| RAD54L | F: AGGCAGGTCCTGTGATGATGA |
| | R:TCAAAGGTTTCCGAAAAGGAGAC |
| POLD1 | F: ATCCAGAACTTCGACCTTCCG |
| | R:ACGGCATTGAGCGTGTAGG |
| EME1 | F: TTCCAGCCTACCTGTCTA |
| | R: TTTCTTTCCTGTCTTCTCA |
| FIBP | F: CTTCCAGATTCCGCCCTCC |
| | R: AGCACCTCCCGAACAAAGG |
| GAPDH | F:  GGAGCGAGATCCCTCCAAAAT |
| | R:  GGCTGTTGTCATACTTCTCATGG |

[a] F, forward primer; R, reverse primer.

**Supplementary Table S2.** Primers Sequences used for ChIP PCR

| Numbers | Sequences (5'--3') |
|---|---|
| #1 | F: CTACAGAGAAAGGACCCACCC<br>R: CGTGAGTGAGGGCGTTTGA |
| #2 | F: TCTTCAAACGCCCTCACTCA<br>R: GAGGGGAAGTGTGTGGGGTA |
| #3 | F: GATCAGTGTCACCTCCTTCCC<br>R: AATGGGACGCAGTAGTCAGA |
| #4 | F: CACCCCGCTCTGACTACTG<br>R: TTTCGAGACCGGAAGTGAGT |
| #5 | F: CCATGCTTTCGATCACTCACTTC<br>R: ACTTCTCCCGCCACTCTTTCA |
| #6 | F: AAGAGTGGCGGGAGAAGTTG<br>R: CTCCTGGACACCTTAGCCAC |
| #7 | F: ACACCGCTCTGCAGAATCTT<br>R: GCAAATCAGGGGAGTGGGAC |
| #8 | F: GTCCCACTCCCCTGATTTGC<br>R: GGCACACAGCTGGCTAAGTA |

F, forward primer; R, reverse primer.
